# Economic inequality in prevalence of underweight and short stature in children and adolescents: the weight disorders survey of the CASPIAN-IV study

**DOI:** 10.20945/2359-3997000000280

**Published:** 2020-08-24

**Authors:** Ramin Heshmat, Mostafa Qorbani, Nafiseh Mozafarian, Shirin Djalalinia, Ali Sheidaei, Morteza Mansourian, Nastaran Hajizadeh, Mohammad Esmaeil Motlagh, Hamid Asayesh, Armita Mahdavi-Gorabi, Roya Kelishadi

**Affiliations:** 1 Tehran University of Medical Sciences Endocrinology and Metabolism Population Sciences Institute Chronic Diseases Research Center Tehran Iran Chronic Diseases Research Center, Endocrinology and Metabolism Population Sciences Institute, Tehran University of Medical Sciences, Tehran, Iran; 2 Alborz University of Medical Sciences Non-communicable Diseases Research Center Karaj Iran Non-communicable Diseases Research Center, Alborz University of Medical Sciences, Karaj, Iran; 2 Tehran University of Medical Sciences Endocrinology and Metabolism Clinical Sciences Institute Endocrinology and Metabolism Research Center Tehran Iran Endocrinology and Metabolism Research Center, Endocrinology and Metabolism Clinical Sciences Institute, Tehran University of Medical Sciences, Tehran, Iran; 4 Isfahan University of Medical Sciences Research Institute for Primordial Prevention of Non-communicable Disease Child Growth and Development Research Center Isfahan Iran Department of Pediatrics, Child Growth and Development Research Center, Research Institute for Primordial Prevention of Non-communicable Disease, Isfahan University of Medical Sciences, Isfahan, Iran; 5 Ministry of Health and Medical Education Deputy of Research and Technology Development of Research & Technology Center Tehran Iran Development of Research & Technology Center, Deputy of Research and Technology, Ministry of Health and Medical Education, Tehran, Iran; 6 Shahid Beheshti University of Medical Science Department of Epidemiology and Biostatistics Tehran Iran Department of Epidemiology and Biostatistics, Shahid Beheshti University of Medical Science, Tehran, Iran; 7 Iran University of Medical Sciences Health Management and Economics Research Center Tehran Iran Health Management and Economics Research Center, Iran University of Medical Sciences, Tehran, Iran; 8 Ahvaz Jundishapur University of Medical Sciences Department of Pediatrics Ahvaz Iran Department of Pediatrics, Ahvaz Jundishapur University of Medical Sciences, Ahvaz, Iran; 9 Qom University of Medical Sciences Department of Medical Emergencies Qom Iran Department of Medical Emergencies, Qom University of Medical Sciences, Qom, Iran; 10 Tehran University of Medical Sciences Tehran Heart Center Department of Basic and Clinical Research Tehran Iran Department of Basic and Clinical Research, Tehran Heart Center, Tehran University of Medical Sciences, Tehran, Iran

**Keywords:** Underweight, short stature, inequality, socio-economic factors, Iran

## Abstract

**Objective:**

The aim of this study was to determine the determinants of socio-economic inequality in the prevalence of short stature and underweight in Iranian children and adolescents.

**Subjects and methods:**

This cross-sectional nationwide study was conducted on 36,486 participants, aged 6-18 years. This school-based surveillance (CASPIAN- IV) program and its complementary part on weight disorders evaluation was conducted in urban and rural areas of 30 provinces in Iran. In addition to physical examination, a validated questionnaire was completed from students and their parents. Socio-economic status (SES) was determined using principal component analysis, and was classified in quintile scale. Inequality in the prevalence of underweight and short stature was assessed using concentration (C) index and slop index of inequality (SII) by the Oaxaca-Blinder decomposition method.

**Results:**

The prevalence (95% CI) of underweight and short stature at national level was 10.89 (10.55, 11.23) and 4.15 (3.94, 4.38), respectively; it had a downtrend from the lowest to highest SES quintile. Furthermore, the value of C for underweight and short stature was negative, i.e. inequality was in favor of high SES groups. Moreover, the prevalence gap of underweight and short stature in the first and fifth quintiles of SES was 6.58% and 5.80%, respectively. The highest proportion of this gap was explained by living area. In the multiple logistic model, odds of underweight and short stature were significantly lower in individuals with higher SES. Compared to boys, odds of underweight were decreased in girls, whereas odds of short stature were increased in them. Odds of underweight and short stature were increased in participants from rural areas than in urban areas. With increasing age, the odds of underweight and short stature decreased significantly.

**Conclusions:**

The results of this study showed that inequality in the prevalence of short stature and underweight was in favor of high SES groups. Moreover, living area was one of the most important determinants that explained this inequality. Therefore, this issue needs to be considered in health promotion policies.

## INTRODUCTION

Malnutrition is one of the most important health concerns in developing countries (1–3). Malnutrition in early life leads to physical growth retardation, underweight and short stature, associated with the frequent infections and resistance to treatment. In addition to reducing physical growth, emotional disorders, negative impact on learning and reducing the work efficiency may also be more common in children suffering from malnutrition. In the coming years, these children will not be able to reach age-appropriate mental and physical abilities (4,5).

Weight status is influenced by multiple underlying factors like complicated gene-environment Interactions. Socioeconomic status (SES) includes social environment, social norms and media exposure, which can affect diet and body weight (3). According to WHO report, in developing countries, about 183 million children are underweight and 226 million are short stature (6). The overall prevalence of stunting, underweight, and wasting in Guatemala in 2006 was 34.4%, 7.6%, and 0.8%, respectively (7). The prevalence of underweight, short stature, and wasting among Indianan children less than five years in 2013 was 33%, 40%, and 17%, respectively (8). According to another cross-sectional study, underweight was 17.5% and the prevalence of short stature was 2.6% in Iranian children (9).

Although the prevalence of child malnutrition in developing countries was reduced (10), SES inequality in malnutrition was observed especially in developing countries and some studies confirmed SES inequality (11–13). A cross-sectional study conducted on Guatemalan children showed that SES inequality in short stature is to the detriment of poor families (12). Another cross-sectional study conducted on Colombian children and adolescents showed that children and adolescents in poor families are five times more likely to be shorter than affluent families (13).

Moreover, in some studies, Oaxaca-Blinder decomposition method was used to determine the causes of inequality in malnutrition (8,14–17). For example, a cross-sectional study conducted on Indian children less than five years demonstrated that malnutrition rate was higher in the urban poor families. The main factors contributing to the gap between the high and low SES groups in malnutrition were as follows: lower rate of using health care, the mothers’ low BMI, and low parental educational levels (8). Another cross-sectional study performed in 2014 showed that there were SES inequalities in children's stunning. The C value of this inequality was negative, indicating that the inequality was in favor of the high SES group. In addition, the mothers’ education was the most important factor contributing to the inequality in short stature (17).

There is limited evidence of inequality in short stature and underweight in children and adolescents, and most studies have been conducted on children under five years of age. Therefore, understanding the extent and the factors associated with short stature and underweight in children and adolescents is crucial. This study was aimed to assess SES inequalities and their associated factors in the prevalence of short stature and underweight in Iranian children and adolescents.

## SUBJECTS AND METHODS

The present study was designed based on combined data of national survey of school-based surveillance system of **C**hildhood and **A**dolescence **S**urveillance and **P**revent**I**on of **A**dult **N**on-communicable disease (CASPIAN-IV) study (18) and its complementary part on weight disorders evaluation (19,20).

Through this investigation, 36,486 school students aged 10-18 years living in urban and rural areas of 30 provinces in Iran were randomly selected. Following the protocols of WHO-global school-based student health survey (GSHS-WHO),

Trained research experts followed all processes of examinations and inquiry with calibrated instruments. Information was recorded in the checklists and validated questionnaires for all participants (21). In order to assess the standards of quality of data in multi-center research, all levels of quality assurance were closely supervised and monitored by Data and Safety Monitoring Board (DSMB) of the project (18,21,22).

### Definition of terms

- **Demographic information:** All participants’ demographic information including age, sex, residence area, family characteristics, family history (FH) of obesity, parental level of education, possessing a family private car, type of home etc., were gathered. Some complementary information on screen time (ST), physical activity (PA), and many other components of life styles were also questioned.
- **Socioeconomic status (SES):** Family SES was estimated using previous approach in the Progress in the International Reading Literacy Study (PIRLS) for Iran (23). By principle component analysis (PCA), variables of parents’ education, parents’ job, possessing private car, school type (public/private), type of home (private/rented) and having personal computer in home were combined as one main component of SES (24). This unique scale was categorized into 5 quintiles. Based on that, the first quintile was defined as a “lowest SES” and the fifth quintile as a “highest SES” groups.
- **ST:** The ST was calculated as the sum of the average daily hours spent onwatching television or video, as well as for leisure time use of personal computer (PC) or electronic games (EG). ST was asked separately for week days and weekends. To analyze correlates of ST, according to the international ST recommendations, ST was categorized into two groups: less than 2 hours per day (Low), and 2 hours per day or more (High) (25).
- **PA:** The recalls of activities in the prior week to the study were collected. The participants reported the weekly frequency of their leisure time PA outside the school. For PA, the two following questions were asked: 1) during the past week, on how many days were you physically active for overall 30 minutes per day? Response options were from 0 to 7 days; and 2) How much time do you spend in exercise class regularly in school per week? Responses ranged from 0 to 3 or more hours. PA less than two times per week was considered as mild, two to four times a week was considered as moderate and more than 4 hours a week was considered as vigorous PA (26).

### Measurements

Weight was measured to the nearest 200 g in barefoot and lightly dressed condition. Body mass index (BMI) was calculated as weight in kilograms divided by height in meters squared (m^2^) (18).

Underweight was defined as BMI < 5^th^ percentile for age and sex based on the World Health Organization standard growth curves. Short stature was defined as height less than −2 standard deviation (SD) below the mean height for age and sex (27,28).

After explanation of the study, participants and one of their parents were assured that their responses would remain anonymous and confidential. Participation in the study was voluntary and all of potential participants had the freedom to withdraw from the study at any time. Both written and verbal informed consent was obtained from the study participants and one of their parents respectively.

### Statistical analysis

Continuous variables were presented as mean and SD. Prevalence of underweight and short stature was estimated with a 95% confidence interval (CI). Association of independent variables with underweight and short stature was assessed using univariate and multivariate logistic regression analysis. The results of logistic regression analysis were presented as OR (95% CI).

SES inequality in underweight and short stature was estimated by calculating the prevalence of underweight and short stature across quintiles of SES, the concentration index (C) and slop index of inequality (SII). To assess the association of weight disorders across SES quintiles, we used C which interpreted on the basis of the distribution of target variable versus SES distribution (29,30). The C value was estimated using the following formula: $c=\frac{2}{n\mu }\sum_{i=1}^{n}hiRi-1$. Where *hi* is the amount of each weight disorders for the *i – th* individual, *Ri* is the relative rank of the *i – th* individual in the distribution of the SES variable and µ is the mean value of the weight disorders. The negative and positive values of C showed that inequality was in favor of high and low SES groups of the SES, respectively (29).

Decomposition of the gap in weight disorders between the first and fifth quintiles of SES was investigated using the counterfactual decomposition method (31,32). This method divides the gap between the means of an interested outcome variable into two components. The “explained” (endowment) component arises because of differences in groups’ characteristics, such as differences in age, sex or other characteristics of two groups, and an ‘unexplained’ (coefficient) component is extracted from the differential effects of these characteristics.

Statistical measures were assessed using survey data analysis methods in the STATA software (version 11.1, Stata Corporation, College Station, TX, USA). *P* < 0.05 was considered as statistically significant. Missing data were imputed using Amelia package version 1.7.3 in R statistical package.

## RESULTS

Overall, 36,486 students participated in this survey (response rate: 91.3%). average age of students was 12.14 (3.^36^) years and no statistically significant differences were found between males and females. Considering the sex 49.21% and regarding the resident area 74.23% of participants were aligned to females and urban areas resident, respectively.

Appendix 1 presents the socioeconomic inequality in the prevalence of underweight in Iranian children and adolescents at national and provincial levels. By increasing SES quintiles, values of these variables followed trends with different slopes in various provinces.

The national estimation of prevalence of underweight was 10.89% (95% CI: 10.55, 11.23) with descending changing from [14.61% (13.76, 15.49)] to [8.02% (7.38, 8.72)] according to SES categories. At provincial level, the highest and the lowest prevalence of underweight were [23.21% (20.94, 25.66)] and [4.87% (3.19, 7.37)], respectively. Considering the SII values, at national level [-0.01(-0.10, 0.07)] absolute difference in prevalence of underweight between the bottom and top of the socioeconomic groups showed the descending trends.

At national level, negative C index (-0.12) indicated that inequality was in favor of high SES groups.

Short stature with different patterns among provinces had a national estimation of 4.15[3.94, 4.38] with nearly descending changes along with the SES categories. At provincial level, the highest and the lowest prevalence rates were 8.69 [7.23, 10.39] and 1.20 [0.70, 2.06] respectively. Considering the SII values, at national level 0.06[-0.01, 0.15] the absolute difference in prevalence of short stature between the bottom and top of the socioeconomic groups showed ascending trends. Negative C index at national level (-0.25) suggested that inequality was in favor of high SES groups (Appendix 2).

**Figure 1:**
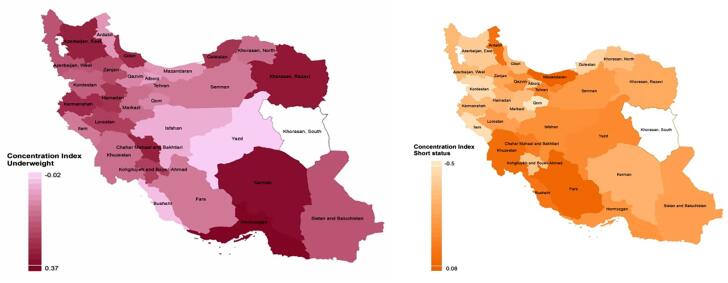
The C index of underweight and short statue at provincial levels in Iranian children and adolescents: the CASPIAN IV study.

**Figure 2:**
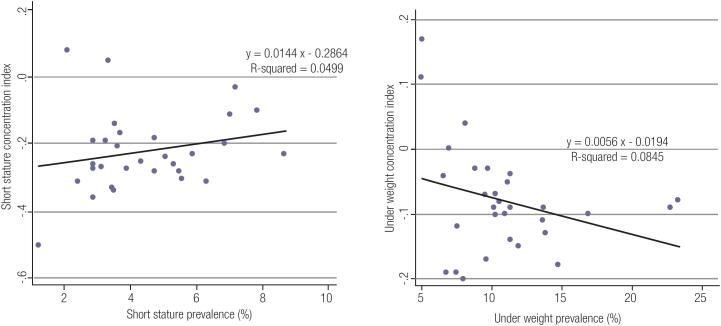
The association of C index with prevalence of underweight and short stature in Iranian children and adolescents: the CASPIAN IV study.

Figure 1 shows the C index of underweight and short statue at provincial levels. Figure 2 shows the association of C index with prevalence of underweight and short stature. Based on the results, C Index could be used to explain 0.08 % of variation of variable of underweight and 0.05% of variable of short stature.

The gap between the low and high SES groups for prevalence of underweight and short stature was 6.58% and 5.80% respectively. In the explained component living area was seen to make highest contribution to the gap between the two SES groups for prevalence of underweight and short stature (Table 1).

**Table 1:** Decomposition of the gap in prevalence of underweight and short stature between the first and fifth quintiles of socioeconomic status in Iran

		Underweight				Short stature			
		Prediction (%)	95% CI		P	Prediction (%)	95% CI		P
Prevalence in first quintile of SES		14.68	13.79	15.57	<0.001	8.53	7.83	9.24	<0.001
Prevalence in fifth quintile of SES		8.09	7.41	8.77	<0.001	2.74	2.33	3.14	<0.001
Differences (Total gap)		6.59	5.47	7.72	<0.001	5.80	4.99	6.61	<0.001
Due to endowments (explained)									
	Physical activity	-0.03	-0.07	0.01	0.177	-0.05	-0.10	0.00	0.055
	Sex	-0.01	-0.04	0.01	0.355	0.01	-0.01	0.03	0.458
	Screen time	0.16	0.00	0.33	0.048	0.43	0.32	0.53	<0.001
	Region	0.96	0.35	1.58	0.002	1.01	0.54	1.47	<0.001
	FH of obesity	0.20	0.11	0.29	<0.001	–	–	–	–
	Age	-0.05	-0.10	-0.01	0.027	-0.05	-0.10	-0.01	0.019
	Subtotal	1.23	0.58	1.89	<0.001	1.34	0.85	1.83	<0.001
Due to coefficients (unexplained)									
	Physical activity	0.07	-0.39	0.53	0.761	-0.46	-0.84	-0.07	0.019
	Sex	-5.42	-8.82	-2.03	0.002	2.19	-0.24	4.62	0.077
	Screen time	-0.81	-1.49	-0.13	0.019	-1.13	-1.53	-0.73	<0.001
	Region	0.65	-3.32	4.62	0.749	1.21	-1.51	3.93	0.382
	FH of obesity	-0.77	-1.55	0.01	0.053	–	–	–	–
	Age	4.24	0.24	8.24	0.038	-4.03	-7.07	-0.99	0.009
	Subtotal	5.36	4.10	6.62	<0.001	4.46	3.58	5.34	<0.001

SES: socioeconomic status: SES is an economic and sociological combined total measure of a person's work experience and of an individual's or family's economic and social position in relation to others. FH: family history: past occurrences (of a medical or mental health condition) in family members or past incidences (of a type of behavior) by family members.

In multivariate logistic regression analysis, odds of underweight and short stature were significantly lower in individual with higher SES (p < 0.001). Compared to males, odds of underweight were decreased in females [OR: 0.86 (95% CI: 0.80, 0.93)], and odds of short stature were increased in them [OR: 1.30 (95% CI: 1.16, 1.46)] (p < 0.001). Participant from rural areas compared to those who were from urban areas had increased odds of underweight [OR: 1.22 (95% CI: 1.12, 1.32)] and short stature [OR: 1.35 (95% CI: 1.20, 1.53)] (p < 0.001). Each year increment in age decrease the odds of underweight [OR: 0.97 (95% CI: 0.96, 0.98)] and short stature [OR: 0.91(95% CI: 0.89, 0.93)] (p < 0.001) (Table 2).

**Table 2:** Association of independent variables with underweight and short stature in multivariate logistic regression

Variables		Underweight		Short stature	
		Crude OR (95% CI)	Adjusted OR (95% CI)	Crude OR (95% CI)	Adjusted OR (95% CI)
SES	(Q1)				
	Q2	0.80 (0.72, 0.89)*	0.83 (0.75, 0.92)*	0.47 (0.40, 0.55)*	0.52 (0.45, 0.61)*
	Q3	0.71 (0.64, 0.79)*	0.76 (0.68, 0.84)*	0.31 (0.26, 0.37)*	0.39 (0.32, 0.46)*
	Q4	0.57 (0.51, 0.64)*	0.62 (0.55, 0.70)*	0.29 (0.24, 0.34)*	0.36 (0.29, 0.43)*
	Q5	0.51 (0.45, 0.57)*	0.56 (0.50, 0.64)*	0.30 (0.25, 0.36)*	0.36 (0.30, 0.44)*
Physical activity	Low				
	Moderate	1.01 (0.94, 1.09)	0.95 (0.88, 1.04)	1.07 (.94, 1.21)	1.03 (.90, 1.18)
	High	1.10 (1.00, 1.22)	1.03 (0.92, 1.15)	2.22 (1.94, 2.56)*	1.88 (1.61, 2.20)*
Sex	Boy				
	Girl	0.86 (0.81, 0.92)*	0.86 (0.80, 0.93)*	1.15 (1.03, 1.28)*	1.30 (1.16, 1.46)*
Screen time	≤2h				
	>2h	0.82 (0.76, 0.88)*	0.91 (0.84, 0.98)*	0.42 (0.36, 0.48)*	0.52 (0.44, 0.60)*
Region	Urban					
	Rural	1.48 (1.38, 1.59)*	1.22 (1.12, 1.32)*	2.14 (1.92, 2.38)*	1.35 (1.20, 1.53)*
FH of obesity	(No)				
	Yes	0.81 (0.75, 0.88)*	0.80 (0.74, 0.87)*	–	–
Age	(year)	0.96 (0.95, 0.97)*	0.97 (0.96, 0.98)*	0.88 (0.87, 0.90)*	0.91 (0.89, 0.93)*

SES: socioeconomic status: SES is an economic and sociological combined total measure of a person's work experience and of an individual's or family's economic and social position in relation to others. FH: family history: past occurrences (of a medical or mental health condition) in family members or past incidences (of a type of behavior) by family members.

* Statistically significant.

## DISCUSSION

This national study showed that the prevalence of underweight and short stature, with different patterns in the provinces and national estimation, was 10.89 [10.55, 11.23] and 4.15 [3.94, 4.38], respectively. Moreover, SES inequalities were documented in the prevalence of underweight and short stature in Iranian children and adolescents. The value of C index for underweight and short stature was negative, showing that the inequality was to the detriment of poor families. The frequency of differences in underweight and short stature between the first and fifth quintiles was 6.59% and 5.80%, respectively. It indicated higher prevalence of short stature and underweight in the lowest SES quintiles. Moreover, the results showed that the rate of short stature in low SES classes was about 3 times more than the upper class, which should be considered by health policy makers, in designing future intervention programs to reduce this inequality.

Regarding SES inequality in malnutrition, the results of present study were consistent with previous studies. A study conducted on children under 5 years old in 47 developing countries on demonstrated that SES inequalities in malnutrition were observed in developing countries. Furthermore, it was shown that the inequality in short stature was higher in Latin America and the Caribbean region, according to the C index (11). Some studies in developing countries reported that the C index of inequality for prevalence of short stature was negative, meaning that inequality was in favor of high SES groups and the prevalence of short stature was higher in children in poor families (8,12–13,17,33).

Across-sectional study (2012) in Columbia showed that children and adolescents in poor families were five times more likely to be stunt than affluent families. Over one-third of socioeconomic inequality in short stature is due to care practices and family characteristics, especially maternal education (13).

The results of a longitudinal study in Brazil (2010), showed that during a period of 33 years (1974-2007) the prevalence of short stature was decreased from 37.1% to 7.1% and SES inequality in stunted children had decreased over time. In this study, living area plays a main role in underweight and short stature inequalities, which may be due to food security and access to healthy food and clean water or sanitary facilities such as cultural beliefs and level of health awareness and access to health care is different in rural areas than in cities (33).

Possibly, health conditions, such as the use of water sanitation in urban areas is better than that in rural areas and these hygienic conditions in urban areas have a positive impact on the prevention of infectious diseases and therefore affect children's nutritional status; Moreover, urban families are less likely to face food insecurity and more ability to provide adequate care for children, including health services. The evidence showed that, in general, children in urban areas have a better nutritional status than those in rural areas (34,35).

Providing the same living conditions in urban and rural areas might be a significant contributor to reducing inequalities. So it is better to provide sanitary facilities in rural areas, such as clean water, health services and health care, education interventions to raise mothers ‘nutrition and health awareness, and struggle against culturally wrong nutritional habits. Another suggestion is to create favorable educational conditions for girls in rural areas which can lead to healthier future generations. Studies have always concluded that the mother's education is of a great importance for health, nutrition and child's survival (36–38). Also, recognizing the social and cultural issues related to family understanding of the nutritional needs for food distribution within the family and performing comprehensive interventions (such as cultural, economic, social and nutritional education), especially in rural areas seems necessary to prevent malnutrition.

Our findings about the significant effect of ST on SES inequality, are in line with the previous studies (39,40). In the present study, the higher SES groups had more ST. It was also shown that the risk of underweight and short stature reduced as the ST increased. Some review studies showed that watching TV was correlated with food habits in children or young adults (41–43) and also it was shown that there was a positive correlation between the time spending on watching TV and energy intake (42,44,45) which can lead to weight disorders.

In the present study, in multivariate model, low SES, male gender, living in rural area and increment in age was associated significantly with higher prevalence of underweight and short stature. This finding is concordant with previous studies (7,46–50). A previous study conducted in Iran showed that the prevalence of underweight in boys was higher than that in girls and malnutrition was correlated with mother's occupation, parents’ education and the number of children (47). A cross-sectional study conducted in Guatemala showed that educational status, child and family care are the most influential factors on short stature (7).

### Strengths and limitations

The limitations of this study were as follows: the cross-sectional nature of the study does not allow us to conclude causal inference and using self-reported data. One of the main strengths of this study was a large sample size which let us to assess the SES inequality at national and provincial levels. Moreover, in this study, the standard and validated questionnaire of WHO was used.

In conclusion, the results of the present study showed that inequality in prevalence of short stature and underweight was in favor of high SES groups and living area was one of the most important determinants that explained this inequality. To minimize inequality, policy makers should attend for this hidden part, and take appropriate policies to deal with this issue. In addition, more attention should be given to the lower SES levels to take necessary measures to reduce the gap between upper and lower SES groups.

## Appendix 1 Socioeconomic inequality in prevalence of underweight in Iranian children and adolescents: the CASPIAN IV study

**Table:**

Province	Q1 [95% CI]	Q2 [95% CI]	Q3 [95% CI]	Q4 [95% CI]	Q5 [95% CI]	Total [95% CI]	SII [95% CI]	C (SE)
Ardebil	8.33 [4.21, 15.82]	9.84 [5.66, 16.55]	9.16 [5.27, 15.46]	8.93 [4.86, 15.83]	7.14 [3.24, 15.03]	8.81 [6.70, 11.50]	0.01 [-0.03, 0.06]	-0.03 (0.07)
Bushehr	10.53 [6.33, 17.01]	9.29 [5.46, 15.35]	9.43 [5.76, 15.07]	3.80 [1.71, 8.21]	4.55 [2.180, 9.24]	7.39 [5.72, 9.51]	0.09 [0.01, 0.17]	-0.19 (0.07)
Ch. Mahal	11.55 [8.14, 16.14]	11.88 [8.47, 16.4]	10.82 [7.62, 15.15]	7.17 [4.67, 10.86]	9.81 [6.48, 14.59]	10.21 [8.66, 12]	0.04 [-0.04, 0.12]	-0.07 (0.05)
East Azar.	3.97 [1.66, 9.21]	3.09 [1.00, 9.19]	9.43 [3.97, 20.8]	2.99 [0.74, 11.22]	6.82 [3.09, 14.4]	4.87 [3.19, 7.37]	-0.03 [-0.19, 0.12]	0.11 (0.12)
Fars	14.21 [9.93, 19.94]	17.56 [13.41, 22.65]	14.78 [11.14, 19.34]	11.63 [8.46, 15.77]	10.04 [6.76, 14.67]	13.67 [11.89, 15.67]	0.07 [-0.02, 0.17]	-0.09 (0.04)
Gilan	8.09 [4.53, 14.03]	7.88 [4.88, 12.49]	10.83 [7.67, 15.08]	7.14 [4.7, 10.71]	4.25 [2.48, 7.18]	7.48 [6.13, 9.11]	0.05 [-0.06, 0.16]	-0.12 (0.05)
Golestan	13.06 [9.76, 17.26]	11.95 [8.82, 16]	7.17 [4.62, 10.97]	7.11 [4.25, 11.65]	5.64 [3.15, 9.91]	9.54 [8.05, 11.27]	0.09 [0.04, 0.14]	-0.17 (0.05)
Hamedan	14.29 [10.38, 19.34]	9.09 [6.50, 12.58]	10.34 [7.32, 14.42]	11.71 [8.09, 16.65]	4.55 [2.29, 8.83]	10.17 [8.63, 11.96]	0.07 [-0.06, 0.21]	-0.10 (0.04)
Hormozgan	27.06 [21.95, 32.86]	21.29 [16.17, 27.49]	21.54 [15.29, 29.45]	17.44 [10.78, 26.97]	14.81 [5.66, 33.52]	22.71 [19.75, 25.97]	0.11 [0.05, 0.18]	-0.09 (0.04)
Ilam	14.84 [11.15, 19.48]	18.43 [13.81, 24.17]	16.26 [11.79, 22.00]	9.83 [6.62, 14.36]	9.65 [6.60, 13.9]	13.63 [11.8, 15.7]	0.09 [-0.07, 0.26]	-0.11 (0.04)
Isfahan	10.31 [5.63, 18.12]	12.64 [8.54, 18.31]	12.34 [9.14, 16.45]	11.34 [8.36, 15.21]	8.88 [6.16, 12.65]	11.10 [9.46, 12.98]	0.03 [-0.04, 0.10]	-0.05 (0.04)
Kerman	21.83 [17.4, 27.02]	16.43 [11.97, 22.12]	16.44 [12.09, 21.96]	15.35 [11, 21.01]	13.21 [9.63, 17.85]	16.82 [14.79, 19.07]	0.10 [0.03, 0.17]	-0.10 (0.04)
Kermanshah	13.83 [10.1, 18.67]	13.07 [9.62, 17.53]	7.53 [4.79, 11.64]	10.64 [7.29, 15.28]	8.68 [5.60, 13.21]	10.9 [9.28, 12.77]	0.06 [-0.04, 0.16]	-0.10 (0.05)
Khorasan	16.88 [11.83, 23.5]	15.87 [11.86, 20.93]	10.28 [7.09, 14.67]	10.81 [7.57, 15.22]	7.61 [4.96, 11.5]	11.88 [10.16, 13.85]	0.12 [0.04, 0.19]	-0.15 (0.04)
Khozestan	14.52 [10.14, 20.36]	10.74 [6.33, 17.65]	14.19 [9.52, 20.63]	11.33 [7.16, 17.49]	3.91 [1.63, 9.06]	11.35 [9.25, 13.85]	0.10 [-0.05, 0.26]	-0.14 (0.05)
Koh&Boyer	12.04 [8.92, 16.06]	7.30 [4.58, 11.43]	9.39 [6.13, 14.11]	11.11 [7.35, 16.45]	7.41 [4.92, 11]	9.48 [7.97, 11.23]	0.03 [-0.09, 0.15]	-0.07 (0.05)
Kordestan	12.53 [9.49, 16.38]	10.23 [7.44, 13.93]	10.79 [7.45, 15.38]	7.94 [4.84, 12.75]	8.27 [4.64, 14.33]	10.44 [8.87, 12.26]	0.05 [0.01, 0.09]	-0.08 (0.05)
Lorestan	7.82 [4.68, 12.79]	7.38 [3.88, 13.59]	4.12 [1.55, 10.5]	4.55 [2.05, 9.77]	11.39 [6.02, 20.5]	6.90 [5.13, 9.21]	-0.02 [-0.18, 0.14]	0.00 (0.09)
Markazi	11.4 [6.72, 18.69]	13.98 [8.28, 22.64]	7.94 [3.331, 17.74]	7.32 [3.31, 15.39]	8.51 [4.30, 16.14]	10.09 [7.61, 13.26]	0.06 [-0.06, 0.18]	-0.09 (0.08)
Mazandaran	5.63 [2.13, 14.09]	2.54 [0.821, 7.60]	3.81 [1.91, 7.44]	3.22 [1.74, 5.88]	7.42 [5.32, 10.25]	5.02 [3.9, 6.44]	-0.04 [-0.17, 0.08]	0.17 (0.07)
Qazvin	13.13 [9.09, 18.6]	11.92 [8.72, 16.09]	10.14 [7.13, 14.22]	11.69 [8.24, 16.33]	9.79 [6.59, 14.3]	11.27 [9.64, 13.13]	0.03 [-0.01, 0.08]	-0.04 (0.04)
Qom	12.61 [7.61, 20.19]	11.42 [7.83, 16.36]	8.12 [5.24, 12.39]	6.25 [3.86, 9.96]	4.55 [2.60, 7.84]	7.93 [6.46, 9.70]	0.11 [0.07, 0.14]	-0.20 (0.06)
Semnan	11.01 [6.35, 18.41]	11.52 [7.46, 17.36]	10.12 [6.98, 14.45]	6.59 [4.19, 10.23]	10.45 [7.59, 14.21]	9.658 [8.07, 11.52]	0.03 [-0.08, 0.14]	-0.03 (0.05)
Sistan& B.	26.53 [23.24, 30.1]	20.73 [16.34, 25.93]	19.41 [14.14, 26.06]	19.63 [13.15, 28.25]	13.95 [6.40, 27.78]	23.21 [20.94, 25.66]	0.11 [0.02, 0.19]	-0.08 (0.03)
Tehran	21.62 [13.68, 32.44]	10.24 [6.03, 16.84]	13.2 [9.53, 18.00]	9.82 [7.02, 13.56]	9.76 [7.27, 13]	11.28 [9.60, 13.2]	0.12 [-0.16, 0.39]	-0.09 (0.05)
West Azar.	12.01 [9.03, 15.81]	3.56 [1.92, 6.49]	5.53 [3.30, 9.13]	4.07 [2.20, 7.39]	6.41 [3.48, 11.51]	6.723 [5.48, 8.23]	0.05 [-0.09, 0.20]	-0.19 (0.06)
Yazd	26.19 [15.1, 41.44]	17.74 [10.08, 29.32]	9.64 [4.88, 18.15]	13.59 [8.20, 21.69]	11.25 [7.19, 17.18]	13.78 [10.88, 17.3]	0.19 [-0.14, 0.51]	-0.13 (0.07)
Zanjan	18.22 [13.88, 23.54]	18.07 [14.23, 22.67]	13.21 [9.72, 17.71]	11.17 [7.39, 16.53]	5.38 [2.94, 9.71]	13.99 [12.16, 16.06]	0.15 [0.06, 0.23]	-0.17 (0.04)
NorthKhorasan	6.64 [4.62, 9.45]	9.94 [7.11, 13.72]	8.00 [4.96, 12.67]	6.70 [3.84, 11.44]	9.87 [6.03, 15.73]	8.08 [6.70, 9.71]	-0.01 [-0.10, 0.07]	0.04 (0.05)
Alborz	5.06 [1.91, 12.74]	5.84 [3.14, 10.47]	10.04 [6.97, 14.25]	5.28 [3.39, 8.13]	5.92 [4.03, 8.62]	6.53 [5.31, 8.01]	0.00 [-0.13, 0.14]	-0.04 (0.06)
Iran (total)	14.61 [13.76, 15.49]	12.07 [11.29, 12.89]	10.86 [10.12, 11.65]	8.88 [8.20, 9.60]	8.02 [7.38, 8.72]	10.89 [10.55, 11.23]	0.08 [0.06, 0.11]	-0.12 (0.01)

## Appendix 2 Socioeconomic inequality in prevalence of short stature in Iranian children and adolescents: the CASPIAN IV study

**Table:**

Province	Q1 [95% CI]	Q2 [95% CI	Q3 [95% CI]	Q4 [95% CI]	Q5 [95% CI]	Total [95% CI]	SII [95% CI]	C (SE)
Ardebil	13.98 [8.28, 22.63]	9.09 [5.10, 15.7]	1.55 [0.39, 6.01]	5.41 [2.44, 11.54]	11.9 [6.51, 20.77]	7.81 [5.82, 10.41]	0.03 [-0.23, 0.29]	-0.10 (0.10)
Bushehr	5.22 [2.51, 10.57]	11.43 [7.11, 17.86]	8.23 [4.83, 13.67]	7.60 [4.36, 12.91]	2.60 [0.98, 6.73]	6.99 [5.36, 9.06]	0.05 [-0.12, .22]	-0.11 (0.06)
Ch. Mahal	6.38 [3.94, 10.16]	3.83 [2.07, 6.98]	0.37 [0.05, 2.61]	4.30 [2.46, 7.43]	2.80 [1.26, 6.11]	3.54 [2.65, 4.70]	0.03 [-0.08, 0.14]	-0.14 (0.09)
East Azar.	11.2 [6.73, 18.06]	8.25 [4.17, 15.67]	1.89 [0.26, 12.3]	1.47 [0.21, 9.78]	3.41 [1.10, 10.08]	6.27 [4.32, 8.99]	0.12 [0.00, 0.24]	-0.31 (0.09)
Fars	5.24 [2.84, 9.46]	1.15 [0.372, 3.52]	3.10 [1.62, 5.86]	2.99 [1.56, 5.65]	4.80 [2.68, 8.47]	3.30 [2.45, 4.44]	-0.01 [-0.10, 0.08]	0.05 (0.09)
Gilan	8.82 [5.08, 14.91]	2.49 [1.04, 5.84]	3.61 [1.95, 6.58]	1.70 [0.71, 4.03]	1.95 [0.88, 4.29]	3.13 [2.28, 4.27]	0.07 [-0.06, 0.20]	-0.27 (0.09)
Golestan	7.69 [5.21, 11.23]	3.15 [1.70, 5.75]	0.76 [0.19, 2.98]	1.52 [0.49, 4.62]	2.56 [1.07, 6.02]	3.421 [2.56, 4.57]	0.06 [-0.04, 0.15]	-0.33 (0.08)
Hamedan	6.72 [4.16, 10.7]	2.56 [1.33, 4.84]	1.03 [0.33, 3.16]	0.90 [0.23, 3.54]	3.41 [1.54, 7.39]	2.82 [2.04, 3.88]	0.04 [-0.06, 0.14]	-0.26 (0.10)
Hormozgan	10.24 [7.06, 14.62]	5.94 [3.4, 10.18]	3.85 [1.61, 8.93]	4.65 [1.75, 11.77]	3.70 [0.52, 22.17]	6.87 [5.21, 9.00]	0.06 [0.01, 0.11]	-0.20 (0.07)
Ilam	6.36 [4.04, 9.87]	2.77 [1.25, 6.02]	2.46 [1.03, 5.79]	0.43 [0.06, 2.98]	1.54 [0.58, 4.05]	2.84 [2.04, 3.95]	0.06 [0, 0.13]	-0.36 (0.09)
Isfahan	6.12 [2.77, 12.98]	4.92 [2.58, 9.19]	1.90 [0.85, 4.17]	2.09 [1.00, 4.32]	2.31 [1.10, 4.77]	2.83 [2.04, 3.92]	0.05 [-0.03, 0.12]	-0.19 (0.10)
Kerman	9.54 [6.62, 13.56]	7.21 [4.39, 11.62]	1.84 [0.69, 4.79]	3.47 [1.66, 7.10]	3.40 [1.78, 6.40]	5.27 [4.13, 6.71]	0.08 [-0.03, 0.20]	-0.26 (0.07)
Kermanshah	7.14 [4.54, 11.06]	4.24 [2.42, 7.32]	3.77 [1.97, 7.08]	2.55 [1.15, 5.57]	1.37 [0.44, 4.17]	3.91 [2.96, 5.15]	0.06 [0.03, 0.10]	-0.27 (0.07)
Khorasan	9.38 [5.73, 14.98]	5.58 [3.33, 9.20]	2.78 [1.33, 5.72]	3.09 [1.55, 6.06]	2.66 [1.27, 5.48]	4.30 [3.28, 5.62]	0.08 [-0.02, 0.17]	-0.25 (0.08)
Khozestan	9.68 [6.18, 14.85]	4.13 [1.73, 9.57]	6.45 [3.50, 11.59]	6.04 [3.17, 11.22]	8.59 [4.82, 14.87]	7.17 [5.52, 9.28]	0.00 [-0.12, 0.13]	-0.03 (0.08)
Koh&Boyer	9.57 [6.81, 13.29]	7.27 [4.56, 11.38]	4.23 [2.21, 7.93]	3.18 [1.43, 6.89]	2.02 [0.91, 4.43]	5.49 [4.36, 6.90]	0.10 [0.07, 0.13]	-0.30 (0.06)
Kordestan	7.26 [4.99, 10.46]	2.33 [1.17, 4.60]	2.48 [1.12, 5.41]	1.06 [0.26, 4.14]	1.50 [0.37, 5.82]	3.48 [2.60, 4.64]	0.06 [-0.01, 0.12]	-0.34 (0.07)
Lorestan	10.8 [6.98, 16.32]	3.33 [1.25, 8.57]	3.13 [1.01, 9.27]	4.65 [2.1, 9.99]	4 [1.29, 11.71]	5.87 [4.24, 8.08]	0.07 [-0.06, 0.19]	-0.23 (0.10)
Markazi	8.86 [4.82, 15.7]	4.30 [1.62, 10.94]	1.54 [0.21, 10.19]	6.10 [2.55, 13.87]	1.06 [0.15, 7.21]	4.70 [3.08, 7.11]	0.08 [-0.06, 0.21]	-0.28 (0.11)
Mazandaran	2.82 [0.70, 10.59]	0	1.91 [0.71, 4.97]	2.57 [1.29, 5.06]	2.25 [1.22, 4.14]	2.08 [1.40, 3.09]	-0.01 [-0.09, 0.07]	0.08 (0.10)
Qazvin	6.15 [3.53, 10.53]	2.99 [1.56, 5.65]	3.15 [1.64, 5.94]	2.82 [1.35, 5.81]	1.71 [0.64, 4.47]	3.24 [2.40, 4.38]	0.04 [-0.01, 0.09]	-0.19 (0.09)
Qom	5.41 [2.45, 11.52]	0.91 [0.23, 3.58]	1.71 [0.64, 4.47]	0	0.38 [0.05, 2.65]	1.20 [0.70, 2.06]	0.05 [-0.04, 0.14]	-0.50 (0.13)
Semnan	9.09 [4.96, 16.09]	4.85 [2.44, 9.40]	3.50 [1.83, 6.60]	1.10 [0.35, 3.34]	3.29 [1.83, 5.85]	3.60 [2.66, 4.85]	0.07 [-0.06, 0.21]	-0.21 (0.10)
Sistan&Blou.	12.58 [10.22, 15.39]	5.09 [3.04, 8.42]	3.49 [1.57, 7.55]	2.80 [0.90, 8.35]	9.52 [3.62, 22.8]	8.69 [7.23, 10.39]	0.07 [-0.09, 0.23]	-0.23 (0.04)
Tehran	8.11 [3.68, 16.91]	3.94 [1.65, 9.12]	4.8 [2.74, 8.27]	3.05 [1.65, 5.58]	2.62 [1.46, 4.67]	3.67 [2.74, 4.90]	0.06 [-0.04, 0.16]	-0.17 (0.08)
West Azar.	5.34 [3.43, 8.22]	2.85 [1.43, 5.59]	0.79 [0.20, 3.11]	2.03 [0.85, 4.79]	1.89 [0.61, 5.69]	2.86 [2.08, 3.92]	0.04 [-0.02, 0.10]	-0.27 (0.09)
Yazd	11.9 [5.03, 25.65]	6.56 [2.47, 16.26]	2.44 [0.61, 9.28]	3.88 [1.46, 9.93]	3.80 [1.71, 8.22]	4.71 [3.09, 7.12]	0.10 [-0.10, 0.30]	-0.18 (0.13)
Zanjan	9.02 [6.01, 13.32]	5.31 [3.33, 8.38]	4.68 [2.73, 7.89]	2.14 [0.80, 5.57]	2.70 [1.13, 6.34]	5.03 [3.93, 6.41]	0.07 [0.02, 0.12]	-0.24 (0.07)
NorthKhorasan	9.72 [7.23, 12.94]	4.06 [2.37, 6.87]	2.5 [1.04, 5.87]	2.25 [0.84, 5.84]	3.97 [1.79, 8.57]	5.43 [4.31, 6.82]	0.07 [-0.02, 0.16]	-0.28 (0.06)
alborz	7.60 [3.45, 15.91]	4.65 [2.34, 9.03]	2.23 [1.00, 4.88]	1.39 [0.58, 3.29]	1.42 [0.64, 3.13]	2.38 [1.68, 3.37]	0.08 [-0.03, 0.18]	-0.31 (0.10)
Iran (total)	8.48 [7.82, 9.20]	4.18 [3.72, 4.7]	2.80 [2.43, 3.24]	2.60 [2.23, 3.02]	2.72 [2.35, 3.15]	4.15 [3.94, 4.38]	0.06 [-0.01, 0.15]	-0.25 (0.01)
